# ﻿Four new species of *Diaporthe* (Diaporthaceae, Diaporthales) from forest plants in China

**DOI:** 10.3897/mycokeys.91.84970

**Published:** 2022-07-06

**Authors:** Lingxue Cao, Dun Luo, Wu Lin, Qin Yang, Xiaojun Deng

**Affiliations:** 1 Key Laboratory for Non-Wood Forest Cultivation and Conservation of the Ministry of Education, Central South University of Forestry and Technology, Changsha 410004, China; 2 Key Laboratory of National Forestry and Grassland Administration for Control of Diseases and Pests of South Plantation, Central South University of Forestry and Technology, Changsha 410004, China; 3 Hunan Provincial Key Laboratory for Control of Forest Diseases and Pests, Central South University of Forestry and Technology, Changsha 410004, China; 4 Guangxi State-owned Bobai Forest Farm, Yulin, Guangxi 537600, China; 5 Guangxi Zhuang Autonomous Region Forestry Research Institute, Nanning 530002, China

**Keywords:** Diaporthaceae, DNA phylogeny, four new taxa, systematics, taxonomy

## Abstract

Species of *Diaporthe* inhabit a wide range of plant hosts as plant pathogens, endophytes and saprobes. During trips to collect forest pathogens in Beijing, Jiangxi, Shaanxi and Zhejiang Provinces in China, 16 isolates of *Diaporthe* were obtained from branch cankers and leaf spots. These isolates were studied by applying a polyphasic approach including morphological, cultural data, and phylogenetic analyses of the nuclear ribosomal internal transcribed spacer (ITS), calmodulin (*cal*), histone H3 (*his3*), partial translation elongation factor-1α (*tef-1α*) and β-tubulin (*tub2*) loci. Results revealed four new taxa, *D.celticola*, *D.meliae*, *D.quercicola*, *D.rhodomyrti***spp. nov.** and two known species, *D.eres* and *D.multiguttulata*.

## ﻿Introduction

*Diaporthe* Nitschkes (syn. *Phomopsis*) is a large genus in the Diaporthaceae with plant pathogens, endophytes or saprobes (Muralli et al. 2006; Rossman et al. 2007; Santos and Phillips 2009; Santos et al. 2011; Udayanga et al. 2011, 2014a, b, 2015; Fan et al. 2015, 2018; Du et al. 2016; Dissanayake et al. 2017; Guarnaccia and Crous 2017, 2018; Guarnaccia et al. 2018; Yang et al. 2018, 2020, 2021a, b; Guo et al. 2020; Sun et al. 2021). Currently, more than 1100 epithets for *Diaporthe* and 950 for *Phomopsis* are listed in Index Fungorum (http://www.indexfungorum.org/; accessed 1 April 2022) with names often based on host association.

The family Diaporthaceae was established by von Höhnel (1917) and was accommodated in the order Diaporthales. Wehmeyer (1975) confined this family to include *Diaporthe* and *Mazzantia*. Later, Diaporthaceae was synonymised under Valsaceae (Barr 1978). However, analysis of LSU sequence data of diaporthalean taxa showed the distinct placement of Diaporthaceae in Diaporthales where it formed a well-supported clade (Castlebury et al. 2002). *Diaporthe*, the type genus of Diaporthaceae, is characterised by immersed ascomata and an erumpent pseudostroma with elongated perithecial necks (Gomes et al. 2013). Asci are unitunicate, clavate to cylindrical. Ascospores are fusoid, ellipsoid to cylindrical, hyaline, biseriate to uniseriate in the ascus, sometimes with appendages (Udayanga et al. 2011). The asexual morph is characterised by ostiolate conidiomata, with cylindrical phialides producing three types (alpha, beta, and gamma conidia) of hyaline, aseptate conidia (Udayanga et al. 2011; Gomes et al. 2013).

In China, the classification of *Diaporthe* has been progressing and the basis for the species identification is a combination of morphological, cultural and phylogenetical analyses (Huang et al. 2015; Gao et al. 2017; Guarnaccia and Crous 2017; Yang et al. 2017, 2018, 2020, 2021a, b; Manawasinghe et al. 2019; Jiang et al. 2021; Huang et al. 2021; Sun et al. 2021; Wang et al. 2021). The present study was conducted to identify *Diaporthe* species that cause dieback and leaf spot disease in Beijing, Jiangxi, Shaanxi and Zhejiang Provinces based on modern taxonomic concepts.

## ﻿Materials and methods

### ﻿Fungal isolation

From 2018 to 2020, sample collections have been ongoing in Beijing, Jiangxi, Shaanxi and Zhejiang Provinces, China (Table 1). Collected samples were taken to the laboratory for isolation and photographed, documented and then kept at 4 °C for further study.

**Table 1.** Isolates and GenBank accession numbers of sequences used in this study.

Species	Isolate	GenBank accession numbers				
		ITS	*cal*	*his3*	*tef-1α*	*tub2*
*Diaportheacaciigena*	CBS 129521*	KC343005	KC343247	KC343489	KC343731	KC343973
*Diaportheacericola*	MFLUCC 17-0956*	KY964224	KY964137	NA	KY964180	KY964074
*Diaportheacerigena*	CFCC 52554*	MH121489	MH121413	MH121449	MH121531	NA
*Diaportheacerigena*	CFCC 52555	MH121490	MH121414	MH121450	MH121532	NA
*Diaportheacuta*	PSCG 047*	MK626957	MK691125	MK726161	MK654802	MK691225
*Diaportheacutispora*	LC6161*	KX986764	KX999274	KX999235	KX999155	KX999195
*Diaporthealangii*	CFCC 52556*	MH121491	MH121415	MH121451	MH121533	MH121573
*Diaporthealangii*	CFCC 52557	MH121492	MH121416	MH121452	MH121534	MH121574
*Diaporthealbosinensis*	CFCC 53066*	MK432659	MK442979	MK443004	MK578133	MK578059
*Diaporthealbosinensis*	CFCC 53067	MK432660	MK442980	MK443005	MK578134	MK578060
*Diaporthealleghaniensis*	CBS 495.72*	KC343007	KC343249	KC343491	KC343733	KC343975
*Diaportheambigua*	CBS 114015*	KC343010	KC343252	KC343494	KC343736	KC343978
*Diaportheampelina*	STE-U 2660	AF230751	AY745026	NA	AY745056	JX275452
*Diaportheamygdali*	CBS 126679*	MH864208	KC343264	KC343506	KC343748	KC343990
*Diaportheamygdali* syn. *D.chongqingensis*	PSCG 435	MK626916	MK691209	MK726257	MK654866	MK691321
*Diaportheamygdali* syn. *D.fusicola*	CGMCC 3.17087	KF576281	KF576233	NA	KF576256	KF576305
*Diaportheamygdali* syn. *D.garethjonesii*	MFLUCC 12-0542a	KT459423	KT459470	NA	KT459457	KT459441
*Diaportheamygdali* syn. *D.kadsurae*	CFCC 52586	MH121521	MH121439	MH121479	MH121563	MH121600
*Diaportheamygdali* syn. *D.kadsurae*	CFCC 52587	MH121522	MH121440	MH121480	MH121564	MH121601
*Diaportheamygdali* syn. *D.mediterranea*	SAUCC194.111	MT822639	MT855718	MT855606	MT855836	MT855951
*Diaportheamygdali* syn. *D.ovoicicola*	CGMCC 3.17093	KF576265	KF576223	NA	KF576240	KF576289
*Diaportheamygdali* syn. *D.sterilis*	CBS 136969	KJ160579	KJ160548	MF418350	KJ160611	KJ160528
*Diaportheamygdali* syn. *D.ternstroemiae*	CGMCC 3.15183	KC153098	NA	NA	KC153089	NA
*Diaportheanacardii*	CBS 720.97*	KC343024	KC343266	KC343508	KC343750	KC343992
*Diaportheangelicae*	CBS 111592*	KC343027	KC343269	KC343511	KC343753	KC343995
*Diaportheapiculata*	CFCC 53068	MK432651	MK442973	MK442998	MK578127	MK578054
*Diaportheapiculata*	CFCC 53069	MK432652	MK442974	MK442999	MK578128	MK578055
*Diaportheaquatic*	IFRDCC 3051*	JQ797437	NA	NA	NA	NA
*Diaporthearctii*	DP0482*	KJ590736	KJ612133	KJ659218	KJ590776	KJ610891
*Diaporthearecae*	CBS 161.64*	KC343032	KC343274	KC343516	KC343758	KC344000
*Diaporthearengae*	CBS 114979*	KC343034	KC343276	KC343518	KC343760	KC344002
*Diaporthearezzoensis*	MFLUCC 15-0127*	MT185503	NA	NA	NA	NA
*Diaportheaseana*	MFLUCC 12-0299a*	KT459414	KT459464	NA	KT459448	KT459432
*Diaportheasheicola*	CBS 136967*	KJ160562	KJ160542	NA	KJ160594	KJ160518
*Diaportheaspalathi*	CBS 117169*	KC343036	KC343278	KC343520	KC343762	KC344004
*Diaportheaustralafricana*	CBS 111886*	KC343038	KC343280	KC343522	KC343764	KC344006
*Diaportheaustraliana*	CBS 146457*	MN708222	NA	NA	MN696522	MN696530
*Diaporthebaccae*	CBS 136972*	KJ160565	MG281695	MF418264	KJ160597	MF418509
*Diaporthebatatas*	CBS 122.21	KC343040	KC343282	KC343524	KC343766	KC344008
*Diaporthebauhiniae*	CFCC 53071*	MK432648	MK442970	MK442995	MK578124	MK578051
*Diaporthebauhiniae*	CFCC 53072	MK432649	MK442971	MK442996	MK578125	MK578052
*Diaporthebeilharziae*	BRIP 54792*	JX862529	NA	NA	JX862535	KF170921
*Diaporthebenedicti*	SBen914*	KM669929	KM669862	NA	KM669785	NA
*Diaporthebetulae*	CFCC 50469*	KT732950	KT732997	KT732999	KT733016	KT733020
*Diaporthebetulae*	CFCC 50470	KT732951	KT732998	KT733000	KT733017	KT733021
*Diaporthebetulicola*	CFCC 51128*	KX024653	KX024659	KX024661	KX024655	KX024657
*Diaporthebetulicola*	CFCC 51129	KX024654	KX024660	KX024662	KX024656	KX024658
*Diaporthebetulina*	CFCC 52560*	MH121495	MH121419	MH121455	MH121537	MH121577
*Diaporthebetulina*	CFCC 52561	MH121496	MH121420	MH121456	MH121538	MH121578
*Diaporthebiconispora*	ZJUD62*	KJ490597	NA	KJ490539	KJ490476	KJ490418
*Diaporthebiguttulata*	ZJUD47*	KJ490582	NA	KJ490524	KJ490461	KJ490403
*Diaporthebohemiae*	CBS 143347*	MG281015	MG281710	MG281361	MG281536	MG281188
*Diaporthebrasiliensis*	CBS 133183*	KC343042	KC343284	KC343526	KC343768	KC344010
*Diaporthecaatingaensis*	URM7486*	KY085927	KY115597	KY115605	KY115603	KY115600
*Diaporthecamelliae-sinensis*	SAUCC194.92*	MT822620	MT855699	MT855588	MT855932	MT855817
*Diaporthecanthi*	CPC 19740*	JX069864	KC843174	NA	KC843120	KC843230
*Diaporthecaryae*	CFCC 52563*	MH121498	MH121422	MH121458	MH121540	MH121580
*Diaporthecaryae*	CFCC 52564	MH121499	MH121423	MH121459	MH121541	MH121581
*Diaporthecassines*	CPC 21916*	KF777155	NA	NA	KF777244	NA
*Diaporthecaulivora*	CBS 127268*	MH864501	KC343287	KC343529	KC343771	KC344013
***Diaporthecelticola***	CFCC 53074*	**MK573948**	**MK574587**	**MK574603**	**MK574623**	**MK574643**
***Diaporthecelticola***	**CFCC 53075**	**MK573949**	**MK574588**	**MK574604**	**MK574624**	**MK574644**
***Diaporthecelticola***	**CFCC 53076**	**MK573950**	**MK574589**	**MK574605**	**MK574625**	**MK574645**
*Diaporthecercidis*	CFCC 52565*	MH121500	MH121424	MH121460	MH121542	MH121582
*Diaporthecercidis*	CFCC 52566	MH121501	MH121425	MH121461	MH121543	MH121583
*Diaporthechamaeropis*	CBS 454.81*	KC343048	KC343290	KC343532	KC343774	KC344016
*Diaporthecharlesworthii*	BRIP 54884m*	KJ197288	NA	NA	KJ197250	KJ197268
*Diaporthechensiensis*	CFCC 52567*	MH121502	MH121426	MH121462	MH121544	MH121584
*Diaporthechensiensis*	CFCC 52568	MH121503	MH121427	MH121463	MH121545	MH121585
*Diaporthechrysalidocarpi*	SAUCC194.35*	MT822563	MT855646	MT855532	MT855760	MT855876
*Diaporthecichorii*	MFLUCC 17-1023*	KY964220	KY964133	NA	KY964176	KY964104
*Diaporthecinnamomi*	CFCC 52569*	MH121504	NA	MH121464	MH121546	MH121586
*Diaporthecinnamomi*	CFCC 52570	MH121505	NA	MH121465	MH121547	MH121587
*Diaporthecissampeli*	CPC 27302*	KX228273	NA	KX228366	NA	KX228384
*Diaporthecitri*	AR3405*	KC843311	KC843157	KJ420881	KC843071	KC843187
*Diaporthecitri*	CFCC 53079	MK573940	MK574579	MK574595	MK574615	MK574635
*Diaporthecitriasiana*	CGMCC 3.15224*	JQ954645	KC357491	KJ490515	JQ954663	KC357459
*Diaporthecitrichinensis*	CGMCC 3.15225*	JQ954648	KC357494	KJ420880	JQ954666	KJ490396
*Diaporthecollariana*	MFLU 17-2770*	MG806115	MG783042	NA	MG783040	MG783041
*Diaporthecompactum*	LC3083*	KP267854	NA	KP293508	KP267928	KP293434
*Diaportheconica*	CFCC 52571*	MH121506	MH121428	MH121466	MH121548	MH121588
*Diaportheconica*	CFCC 52572	MH121507	MH121429	MH121467	MH121549	MH121589
*Diaportheconstrictospora*	CGMCC 3.20096*	MT385947	MT424718	MW022487	MT424682	MT424702
*Diaportheconvolvuli*	CBS 124654	KC343054	KC343296	KC343538	KC343780	KC344022
*Diaporthecoryli*	CFCC 53083*	MK432661	MK442981	MK443006	MK578135	MK578061
*Diaporthecoryli*	CFCC 53084	MK432662	MK442982	MK443007	MK538176	MK578062
*Diaporthecorylicola*	CFCC 53986*	MW839880	MW836684	MW836717	MW815894	MW883977
*Diaporthecorylicola*	CFCC 53987	MW839867	MW836685	MW836718	MW815895	MW883978
*Diaporthecrotalariae*	CBS 162.33*	MH855395	JX197439	KC343540	GQ250307	KC344024
*Diaporthecrousii*	CAA 823*	MK792311	MK883835	MK871450	MK828081	MK837932
*Diaporthecucurbitae*	DAOM 42078*	KM453210	NA	KM453212	KM453211	KP118848
*Diaporthecuppatea*	CBS 117499	MH863021	KC343299	KC343541	KC343783	KC344025
*Diaporthecynaroidis*	CBS 122676*	KC343058	KC343300	KC343542	KC343784	KC344026
*Diaporthecytosporella*	FAU461	KC843307	KC843141	MF418283	KC843116	KC843221
*Diaporthediospyricola*	CPC 21169*	KF777156	NA	NA	NA	NA
*Diaporthediscoidispora*	ZJUD89*	KJ490624	NA	KJ490566	KJ490503	KJ490445
*Diaporthedorycnii*	MFLUCC 17-1015*	KY964215	NA	NA	KY964171	KY964099
*Diaporthedrenthii*	CBS 146453*	MN708229	NA	NA	MN696526	MN696537
*Diaporthedurionigena*	VTCC 930005*	MN453530	NA	NA	MT276157	MT276159
*Diaportheelaeagni-glabrae*	LC4802*	KX986779	KX999281	KX999251	KX999171	KX999212
*Diaportheendophytica*	CBS 133811*	KC343065	KC343307	KC343549	KC343791	KC344033
*Diaportheeres*	AR5193*	KJ210529	KJ434999	KJ420850	KJ210550	KJ420799
*Diaportheeres*	AR5211	KJ210538	KJ435043	KJ420875	KJ210559	KJ420828
*Diaportheeres*	CBS 587.79	KC343153	KC343395	KC343637	KC343879	KC344121
*Diaportheeres*	CFCC 52575	MH121510	NA	MH121470	MH121552	MH121592
*Diaportheeres*	CFCC 52576	MH121511	MH121432	MH121471	MH121553	MH121593
*Diaportheeres*	CFCC 52577	MH121512	MH121433	MH121472	MH121554	MH121594
*Diaportheeres*	CFCC 52578	MH121513	MH121434	MH121473	MH121555	MH121595
*Diaportheeres*	CFCC 52579	MH121514	NA	MH121474	MH121556	NA
*Diaportheeres*	CFCC 52580	MH121515	NA	MH121475	MH121557	MH121596
*Diaportheeres*	CFCC 52581	MH121516	NA	MH121476	MH121558	MH121597
*Diaportheeres*	CGMCC 3.15181	KC153096	NA	NA	KC153087	KF576312
*Diaportheeres*	CGMCC 3.17081	KF576282	NA	NA	KF576257	KF576306
*Diaportheeres*	CGMCC 3.17089	KF576267	NA	NA	KF576242	KF576291
*Diaportheeres*	DAOM 695742	KU552025	NA	NA	KU552023	KU574615
*Diaportheeres*	MAFF 625034	JQ807469	KJ435023	KJ420868	JQ807418	KJ420819
*Diaportheeres*	MFLU 17-0646	MG828895	MG829274	NA	MG829270	MG843877
*Diaportheeres*	MFLUCC 16-0113	KU557563	KU557611	NA	KU557631	KU557587
*Diaportheeres*	MFLUCC 17-0963	KY964190	KY964116	NA	KY964146	KY964073
*Diaportheeres* syn. *D.alnea*	CBS 146.46	KC343008	KC343250	KC343492	KC343734	KC343976
*Diaportheeres* syn. *D.camptothecicola*	CFCC 51632	KY203726	KY228877	KY228881	KY228887	KY228893
*Diaportheeres* syn. *D.celastrina*	CBS 139.27	KC343047	KC343289	KC343531	KC343773	KC344015
*Diaportheeres* syn. *D.celeris*	CBS 143349	MG281017	MG281712	MG281363	MG281538	MG281190
*Diaportheeres* syn.*D.ellipicola*	CGMCC 3.17084	KF576270	NA	NA	KF576245	KF576294
*Diaportheeres syn. D. neilliae*	CBS 144.27	KC343144	KC343386	KC343628	KC343870	KC344112
*Diaportheeres* syn. *D.pulla*	CBS 338.89	KC343152	KC343394	KC343636	KC343878	KC344120
*Diaportheeres*	**CSUFTCC101**	**ON076564**	**NA**	**ON081664**	**ON081656**	**NA**
*Diaportheeres*	**CSUFTCC102**	**ON076565**	**NA**	**ON081665**	**ON081657**	**NA**
*Diaportheeres*	**CSUFTCC103**	**ON076566**	**NA**	**ON081666**	**ON081658**	**NA**
*Diaportheeucalyptorum*	CBS 132525*	MH305525	NA	NA	NA	NA
*Diaporthefoeniculacea*	CBS 111553*	KC343101	KC343343	KC343585	KC343827	KC344069
*Diaporthefraxini-angustifoliae*	BRIP 54781*	JX862528	NA	NA	JX862534	KF170920
*Diaporthefraxinicola*	CFCC 52582*	MH121517	MH121435	NA	MH121559	NA
*Diaporthefraxinicola*	CFCC 52583	MH121518	MH121436	NA	MH121560	NA
*Diaporthefructicola*	MAFF 246408*	LC342734	LC342738	LC342737	LC342735	LC342736
*Diaporthefulvicolor*	PSCG 051*	MK626859	MK691132	MK726163	MK654806	MK691236
*Diaportheganjae*	CBS 180.91*	KC343112	KC343354	KC343596	KC343838	KC344080
*Diaportheganzhouensis*	CFCC 53087*	MK432665	MK442985	MK443010	MK578139	MK578065
*Diaportheganzhouensis*	CFCC 53088	MK432666	MK442986	MK443011	MK578140	MK578066
*Diaporthegoulteri*	BRIP 55657a*	KJ197290	NA	NA	KJ197252	KJ197270
*Diaporthegrandiflori*	SAUCC194.84*	MT822612	MT855691	MT855580	MT855809	MT855924
*Diaportheguangxiensis*	JZB320087	MK335765	MK736720	NA	MK500161	MK523560
*Diaporthegulyae*	BRIP 54025	JF431299	NA	NA	JN645803	KJ197271
*Diaportheguttulata*	CGMCC 3.20100*	MT385950	MW022470	MW022491	MT424685	MT424705
*Diaporthehelianthi*	CBS 592.81*	KC343115	KC343357	KC343599	KC343841	KC344083
*Diaportheheliconiae*	SAUCC194.77*	MT822605	MT855684	MT855573	MT855802	MT855917
*Diaportheheterophyllae*	CPC 26215*	MG600222	MG600218	MG600220	MG600224	MG600226
*Diaportheheterostemmatis*	SAUCC194.85*	MT822613	MT855692	MT855581	MT855810	MT855925
*Diaporthehickoriae*	CBS 145.26*	KC343118	KC343360	KC343620	KC343844	KC344086
*Diaporthehispaniae*	CBS 143351*	MG281123	MG281820	MG281471	MG281644	MG281296
*Diaporthehongkongensis*	CBS 115448*	KC343119	KC343361	KC343603	KC343845	KC344087
*Diaporthehubeiensis*	JZB320123*	MK335809	MK500235	NA	MK523570	MK500148
*Diaportheincomplete*	LC6754*	KX986794	KX999289	KX999265	KX999186	KX999226
*Diaportheinconspicua*	CBS 133813*	KC343123	KC343365	KC343607	KC343849	KC344091
*Diaportheinfecunda*	CBS 133812*	KC343126	KC343368	KC343610	KC343852	KC344094
*Diaportheirregularis*	CGMCC 3.20092*	MT385951	MT424721	NA	MT424686	MT424706
*Diaportheisoberliniae*	CPC 22549*	KJ869190	NA	NA	NA	KJ869245
*Diaporthejuglandicola*	CFCC 51134*	KU985101	KX024616	KX024622	KX024628	KX024634
*Diaporthekochmanii*	BRIP 54033*	JF431295	NA	NA	JN645809	NA
*Diaporthekongii*	BRIP 54031*	JF431301	NA	NA	JN645797	KJ197272
*Diaporthekrabiensis*	MFLUCC 17-2481*	MN047100	NA	NA	MN433215	MN431495
*Diaporthelenispora*	CGMCC 3.20101*	MT385952	MW022472	MW022493	MT424687	MT424707
*Diaporthelitchicola*	BRIP 54900*	JX862533	NA	NA	JX862539	KF170925
*Diaporthelitchi*	SAUCC194.22*	MT822550	MT855635	MT855519	MT855747	MT855863
*Diaporthelithocarpi*	CGMCC 3.15175*	KC135104	KF576235	NA	KC153095	KF576311
*Diaporthelongicolla*	FAU599	KJ590728	KJ612124	KJ659188	KJ590767	KJ610883
*Diaporthelongispora*	CBS 194.36*	MH855769	KC343377	KC343619	KC343861	KC344103
*Diaporthelusitanicae*	CBS 123212*	MH863279	KC343378	KC343620	KC343862	KC344104
*Diaporthelutescens*	SAUCC194.36*	MT822564	MT855647	MT855533	MT855761	MT855877
*Diaporthemacadamiae*	CBS 146455*	MN708230	NA	NA	MN696528	MN696539
*Diaporthemacintoshii*	BRIP 55064a*	KJ197289	NA	NA	KJ197251	KJ197269
*Diaporthemalorum*	CAA 734*	KY435638	KY435658	KY435648	KY435627	KY435668
*Diaporthemarina*	MFLU 17-2622*	MN047102	NA	NA	NA	NA
*Diaporthemasirevicii*	BRIP 54256*	KJ197276	NA	NA	KJ197238	KJ197256
*Diaporthemayteni*	CBS 133185*	KC343139	KC343381	KC343623	KC343865	KC344107
*Diaporthemaytenicola*	CPC 21896*	KF777157	NA	NA	NA	KF777250
*Diaporthemelastomatis*	SAUCC194.55*	MT822583	MT855664	MT855551	MT855780	MT855896
*Diaporthemelonis*	CBS 435.87	KC343141	KC343383	KC343625	KC343867	KC344109
***Diaporthemeliae***	CFCC 53089*	**MK432657**	**NA**	**ON081662**	**ON081654**	**MK578057**
***Diaporthemeliae***	**CFCC 53090**	**MK432658**	**NA**	**ON081663**	**ON081655**	**MK578058**
*Diaporthemiddletonii*	BRIP 54884e*	KJ197286	NA	NA	KJ197248	KJ197266
*Diaportheminima*	CGMCC 3.20097*	MT385953	MT424722	MW022496	MT424688	MT424708
*Diaportheminusculata*	CGMCC 3.20098*	MT385957	MW022475	MW022499	MT424692	MT424712
*Diaporthemiriciae*	BRIP 54736j*	KJ197282	NA	NA	KJ197244	KJ197262
*Diaporthemultigutullata*	CFCC 53095	MK432645	MK442967	MK442992	MK578121	MK578048
*Diaporthemultigutullata*	CFCC 53096	MK432646	MK442968	MK442993	MK578122	MK578049
*Diaporthemultigutullata*	**CFCC 53098**	**MK573957**	**MK574592**	**MK574612**	**MK574632**	**MK574652**
*Diaporthemultigutullata*	**CFCC 53099**	**MK573958**	**MK574593**	**MK574613**	**MK574633**	**MK574653**
*Diaporthemultigutullata*	**CFCC 53100**	**MK573959**	**MK574594**	**MK574614**	**MK574634**	**MK574654**
*Diaporthemusigena*	CBS 129519*	KC343143	KC343385	KC343267	KC343869	KC344111
*Diaporthemyracrodruonis*	URM 7972	MK205289	MK205290	NA	MK213408	MK205291
*Diaportheneoarctii*	CBS 109490*	KC343145	KC343387	KC343629	KC343871	KC344113
*Diaportheneoraonikayaporum*	MFLUCC 14-1136*	KU712449	KU749356	NA	KU749369	KU743988
*Diaporthenothofagi*	BRIP 54801*	JX862530	NA	NA	JX862536	KF170922
*Diaporthenovem*	CBS 127269	KC343155	KC343397	KC343639	KC343881	KC344123
*Diaportheocoteae*	CPC 26217*	KX228293	NA	NA	NA	KX228388
*Diaportheoraccinii*	LC3166*	KP267863	NA	KP293517	KP267937	KP293443
*Diaportheovalispora*	ZJUD93*	KJ490628	NA	KJ490570	KJ490507	KJ490449
*Diaportheoxe*	CBS 133186*	KC343164	KC343406	KC343648	KC343890	KC344132
*Diaporthepadina*	CFCC 52590*	MH121525	MH121443	MH121483	MH121567	MH121604
*Diaporthepadina*	CFCC 52591	MH121526	MH121444	MH121484	MH121568	MH121605
*Diaporthepandanicola*	MFLUCC 17-0607*	MG646974	NA	NA	NA	MG646930
*Diaportheparanensis*	CBS 133184*	KC343171	KC343413	KC343655	KC343897	KC344139
*Diaportheparapterocarpi*	CPC 22729	KJ869138	NA	NA	NA	KJ869248
*Diaportheparvae*	PSCG 035	MK626920	MK691169	MK726211	MK654859	MK691249
*Diaporthepascoei*	BRIP 54847*	JX862538	NA	NA	JX862538	KF170924
*Diaporthepassiflorae*	CPC 19183*	JX069860	KY435644	KY435654	KY435623	KY435674
*Diaporthepassifloricola*	CPC 27480*	KX228292	NA	KX228367	NA	KX228387
*Diaporthepenetriteum*	LC3215	KP267879	NA	KP293532	KP267953	NA
*Diaportheperjuncta*	CBS 109745*	KC343172	KC343414	KC343656	KC343898	KC344140
*Diaportheperseae*	CBS 151.73	KC343173	KC343415	KC343657	KC343899	KC343141
*Diaporthepescicola*	MFLUCC 16-0105*	KU557555	KU557603	NA	KU557623	KU557579
*Diaporthephaseolorum*	AR4203*	KJ590738	KJ612135	KJ659220	KJ590739	KJ610893
*Diaporthephillipsii*	CAA 817*	MK792305	MK883831	MK871445	MK828076	MN000351
*Diaporthepodocarpi-macrophylli*	LC6155*	KX986774	KX999278	KX999246	KX999167	KX999207
*Diaporthepometiae*	SAUCC194.72*	MT822600	MT855679	MT855568	MT855797	MT855912
*Diaporthepseudoalnea*	CFCC 54190*	MZ727037	MZ753468	MZ781302	MZ816343	MZ753487
*Diaporthepseudomangiferae*	CBS 101339*	KC343181	KC343423	KC343665	KC343907	KC344149
*Diaporthepseudophoenicicola*	CBS 176.77	KC343183	KC343425	KC343667	KC343909	KC344151
*Diaporthepseudotsugae*	MFLU 15-3228*	KY964225	KY964138	NA	KY964181	KY964108
*Diaporthepsoraleae*	CPC 21634*	KF777158	NA	NA	KF777245	KF777251
*Diaporthepsoraleae-pinnatae*	CPC 21638*	KF777159	NA	NA	NA	KF777252
*Diaporthepterocarpi*	MFLUCC 10-0575*	JQ619901	JX197453	NA	JX275418	NA
*Diaporthepterocarpicola*	MFLUCC 10-0580a*	JQ619887	JX197433	NA	JX275403	JX275441
*Diaporthepungensis*	SAUCC194.112*	MT822640	MT855719	MT855607	MT855837	MT855952
*Diaporthepyracanthae*	CAA483	KY435635	KY435656	KY435645	KY435625	KY435666
***Diaporthequercicola***	CSUFTCC104*	**ON076567**	**ON081670**	**ON081667**	**ON081659**	**NA**
***Diaporthequercicola***	**CSUFTCC105**	**ON076568**	**ON081671**	**ON081668**	**ON081660**	**NA**
***Diaporthequercicola***	**CSUFTCC106**	**ON076569**	**ON081672**	**ON081669**	**ON081661**	**NA**
*Diaportheracemosae*	CPC 26646*	MG600223	MG600219	MG600221	MG600225	MG600227
*Diaportheraonikayaporum*	CBS 133182*	KC343188	KC343430	KC343672	KC343914	KC344156
*Diaportheravennica*	MFLUCC 16-0997	NA	NA	NA	MT394670	NA
***Diaportherhodomyrti***	CFCC 53101*	**MK432643**	**MK442965**	**MK442990**	**MK578119**	**MK578046**
***Diaportherhodomyrti***	**CFCC 53102**	**MK432644**	**MK442966**	**MK442991**	**MK578120**	**MK578047**
*Diaportherhusicola*	CPC 18191*	JF951146	KC843124	NA	KC843100	KC843205
*Diaportherosae*	MFLUCC 17-2658*	MG828894	MG829273	NA	NA	MG843878
*Diaportherosiphthora*	COAD 2914	MT311197	MT313691	NA	MT313693	NA
*Diaportherossmaniae*	CAA 762*	MK792290	MK883822	MK871432	MK828063	MK837914
*Diaportherostrata*	CFCC 50062*	KP208847	KP208849	KP208851	KP208853	KP208855
*Diaportherostrata*	CFCC 50063	KP208848	KP208850	KP208852	KP208854	KP208856
*Diaportherudis*	AR3422	KC843331	KC843146	NA	KC843090	KC843177
*Diaporthesaccarata*	CBS 116311*	KC343190	KC343432	KC343674	KC343916	KC344158
*Diaporthesackstonii*	BRIP 54669b*	KJ197287	NA	NA	KJ197249	KJ197267
*Diaporthesalicicola*	BRIP 54825*	JX862531	NA	NA	JX862537	KF170923
*Diaporthesambucusii*	CFCC 51986*	KY852495	KY852499	KY852503	KY852507	KY852511
*Diaporthesambucusii*	CFCC 51987	KY852496	KY852500	KY852504	KY852508	KY852512
*Diaportheschimae*	CFCC 53103*	MK442640	MK442962	MK442987	MK578116	MK578043
*Diaportheschimae*	CFCC 53104	MK442641	MK442963	MK442988	MK578117	MK578044
*Diaportheschini*	CBS 133181*	KC343191	KC343433	KC343675	KC343917	KC344159
*Diaportheschisandrae*	CFCC 51988*	KY852497	KY852501	KY852505	KY852509	KY852513
*Diaportheschisandrae*	CFCC 51989	KY852498	KY852502	KY852506	KY852510	KY852514
*Diaportheschoeni*	MFLU 15-1279*	KY964226	KY964139	NA	KY964182	KY964109
*Diaporthesclerotioides*	CBS 296.67*	MH858974	KC343435	KC343677	KC343919	KC344161
*Diaporthesearlei*	CBS 146456*	MN708231	NA	NA	NA	MN696540
*Diaporthesennae*	CFCC 51636*	KY203724	KY228875	NA	KY228885	KY228891
*Diaporthesennae*	CFCC 51637	KY203725	KY228876	NA	KY228886	KY228892
*Diaporthesennicola*	CFCC 51634*	KY203722	KY228873	KY228879	KY228883	KY228889
*Diaporthesennicola*	CFCC 51635	KY203723	KY228874	KY228880	KY228884	KY228890
*Diaportheserafiniae*	BRIP 55665a*	KJ197274	NA	NA	KJ197236	KJ197254
*Diaportheshaanxiensis*	CFCC 53106*	MK432654	MK442976	MK443001	MK578130	NA
*Diaportheshaanxiensis*	CFCC 53107	MK432655	MK432977	MK432002	MK578131	NA
*Diaporthesiamensis*	MFLUCC 10-0573a*	JQ619879	JX197423	NA	JX275393	JX275429
*Diaporthesilvicola*	CFCC 54191*	MZ727041	MZ753472	MZ753481	MZ816347	MZ753491
*Diaporthesojae*	FAU635	KJ590719	KJ612116	KJ659208	KJ590762	KJ610875
*Diaporthespartinicola*	CPC 24951*	KR611879	NA	KR857696	NA	KR857695
*Diaporthespinosa*	PSCG 383*	MK626849	MK691129	MK726156	MK654811	MK691234
*Diaporthestictica*	CBS 370.54	KC343212	KC343454	KC343696	KC343938	KC344180
*Diaporthesubclavata*	ZJUD95*	KJ490630	NA	KJ490572	KJ490509	KJ490451
*Diaporthesubcylindrospora*	KUMCC 17-0151	MG746629	NA	NA	MG746630	MG746631
*Diaporthesubellipicola*	KUMCC 17-0153*	MG746632	NA	NA	MG746633	MG746634
*Diaporthesubordinaria*	CBS 464.90	KC343214	KC343456	KC343698	KC343940	KC344182
*Diaporthetaoicola*	MFLUCC 16-0117*	KU557567	NA	NA	KU557635	KU557591
*Diaporthetectonae*	MFLUCC 12-0777*	KU712430	KU749345	NA	KU749359	KU743977
*Diaporthetectonendophytica*	MFLUCC 13-0471*	KU712439	KU749354	NA	KU749367	KU743986
*Diaporthetectonigena*	LC6512	KX986782	KX999284	KX999254	KX999174	KX999214
*Diaportheterebinthifolii*	CBS 133180*	KC343216	KC343458	KC343700	KC343942	KC344184
*Diaporthethunbergii*	MFLUCC 10-0576a*	JQ619893	JX197440	NA	JX275409	JX275449
*Diaporthethunbergiicola*	MFLUCC 12-0033*	KP715097	NA	NA	KP715098	NA
*Diaporthetibetensis*	CFCC 51999*	MF279843	MF279888	MF279828	MF279858	MF279873
*Diaporthetibetensis*	CFCC 52000	MF279844	MF279889	MF279829	MF279859	MF279874
*Diaporthetorilicola*	MFLUCC 17-1051*	KY964212	KY964127	NA	KY964168	KY964096
*Diaporthetoxica*	CBS 534.93*	KC343220	KC343462	KC343704	KC343946	KC344188
*Diaporthetulliensis*	BRIP 62248a*	KR936130	NA	NA	KR936133	KR936132
*Diaportheueckerae*	FAU656*	KJ590726	KJ612122	KJ659215	KJ590747	KJ610881
*Diaportheukurunduensis*	CFCC 52592*	MH121527	MH121445	MH121485	MH121569	NA
*Diaportheukurunduensis*	CFCC 52593	MH121528	MH121446	MH121486	MH121570	NA
*Diaportheundulate*	LC6624*	KX986798	NA	KX999269	KX999190	KX999230
*Diaportheunshiuensis*	CFCC 52594	MH121529	MH121447	MH121487	MH121571	MH121606
*Diaportheunshiuensis*	CFCC 52595	MH121530	MH121448	MH121488	MH121572	MH121607
*Diaporthevaccinii*	CBS 160.32*	KC343228	KC343470	KC343712	KC343954	KC343196
*Diaporthevangueriae*	CBS 137985*	KJ869137	NA	NA	NA	KJ869247
*Diaporthevawdreyi*	BRIP 57887a*	KR936126	NA	NA	KR936129	KR936128
*Diaporthevelutina*	LC4421	KX986790	NA	KX999261	KX999182	KX999223
*Diaportheverniciicola*	CFCC 53109*	MK573944	MK574583	MK574599	MK574619	MK574639
*Diaportheverniciicola*	CFCC 53110	MK573945	MK574584	MK574600	MK574620	MK574640
*Diaportheviniferae*	JZB320071	MK341551	MK500119	NA	MK500107	MK500112
*Diaporthevirgiliae*	CMW 40748	KP247556	NA	NA	NA	KP247575
*Diaporthexishuangbanica*	LC6707*	KX986783	NA	KX999255	KX999175	KX999216
*Diaporthexunwuensis*	CFCC 53085*	MK432663	MK442983	MK443008	MK578137	MK578063
*Diaporthexunwuensis*	CFCC 53086	MK432664	MK442984	MK443009	MK578138	MK578064
*Diaportheyunnanensis*	LC6168*	KX986796	KX999290	KX999267	KX999188	KX999228
*Diaporthezaobaisu*	PSCG 031*	MK626922	NA	MK726207	MK654855	MK691245
*Diaporthellacorylina*	CBS 121124*	KC343004	KC343246	KC343488	KC343730	KC343972

Strains in this study are marked in bold. NA: Not available. Ex-type/ex-epitype isolates are marked by *.

A total of 16 isolates from host material were obtained by removing a mucoid conidia mass from conidiomata, spreading the suspension on the surface of 1.8% potato dextrose agar (PDA), and incubating at 25 °C for up to 24 h. Single germinating conidium was removed and plated onto fresh PDA plates. Specimens were deposited in the Museums of the Beijing Forestry University ( BJFC) and Central South University of Forestry and Technology ( CSUFT). Axenic cultures were maintained in the China Forestry Culture Collection Centre ( CFCC) and Central South University of Forestry and Technology Culture Collection ( CSUFTCC).

### ﻿Morphological and cultural characterization

Agar plugs (6 mm diam) were taken from the edge of actively growing cultures on PDA and transferred onto the centre of 9 cm diam Petri dishes containing 2% tap water agar supplemented with sterile pine needles (PNA) (Smith et al. 1996) and potato dextrose agar (PDA) and incubated at 25 °C under a 12 h near-ultraviolet light/12 h dark cycle to induce sporulation as described in recent studies (Gomes et al. 2013; Lombard et al. 2014). Colony characters and pigment production on PNA and PDA were noted in the 10-day culture. Colony features were rated according to the color charts of Rayner (1970). Cultures were examined periodically for the development of conidiomata. The microscopic examination was based on the morphological features of conidiomata obtained from the fungal growth mounted in clear lactic acid. At least 30 conidiomata and conidia were measured to calculate the mean size/length. Micro-morphological observations were done at ×1000 magnification using a Leica compound microscope (DM 2500) with interference contrast (DIC) optics. Descriptions, nomenclature, and illustrations of taxonomic novelties were deposited at MycoBank (www.MycoBank.org) (Crous et al. 2004).

### ﻿DNA extraction, PCR amplification and sequencing

Genomic DNA was extracted from colonies grown on cellophane-covered PDA using a CTAB [cetyltrimethylammonium bromide] method (Doyle and Doyle 1990). For PCR amplifications of phylogenetic markers, five different primer pairs were used (Yang et al. 2018). The PCR conditions were: an initial denaturation step of 8 min at 95 °C followed by 35 cycles of 30 s at 95 °C, 30 s at 51 °C (ITS), 58 °C (*his3*) or 55 °C (*cal*, *tef-1α*, *tub2*), and 1 min at 72 °C, and a final elongation step of 5 min at 72 °C. PCR amplification products were assayed via electrophoresis in 2% agarose gels. DNA sequencing was performed using an ABI PRISM 3730XL DNA Analyzer with a BigDye Terminater Kit v.3.1 (Invitrogen, Waltham, MA, USA) at the Shanghai Invitrogen Biological Technology Company Limited (Beijing, China).

### ﻿Phylogenetic analyses

The quality of our amplified nucleotide sequences was checked and combined by SeqMan v.7.1.0 and reference sequences (Table 1) were retrieved from the National Center for Biotechnology Information (NCBI), according to recent publications of the genus (Guo et al. 2020; Sun et al. 2021; Yang et al. 2021b). Sequences were aligned using MAFFT v. 6 (Katoh and Toh 2010) and manually corrected using Bioedit 7.0.9.0 (Hall 1999). Phylogenetic analyses were carried out with maximum likelihood analysis (ML), which was performed at the CIPRES web portal (Miller et al. 2010), 1000 rapid bootstrap replicates were run with GTRGAMMA model of nucleotide evolution. Bayesian inference analysis (BI) was performed in MrBayes v. 3.2.0 (Ronquist and Huelsenbeck 2003). The best-fit nucleotide substitution models for each gene were selected using jModelTest v. 2.1.7 (Darriba et al. 2012) under the Akaike Information Criterion. The best nucleotide substitution model for ITS, *his3* and *tub2* was TrN+I+G, while HKY+I+G was selected for both *cal* and *tef-1α*. Phylogenetic trees were viewed in FigTree v1.4. The names of the isolates from the present study are marked in blue in the trees. Maximum likelihood bootstrap support values ≥ 75% (BT) are given at the nodes. Bayesian posterior probabilities ≥ 0.95 (PP) were thickened in the phylogenetic tree. Alignment and trees were deposited in TreeBASE (submission ID: S29621).

## ﻿Results

### ﻿Phylogenetic analyses

**Figure 1.**
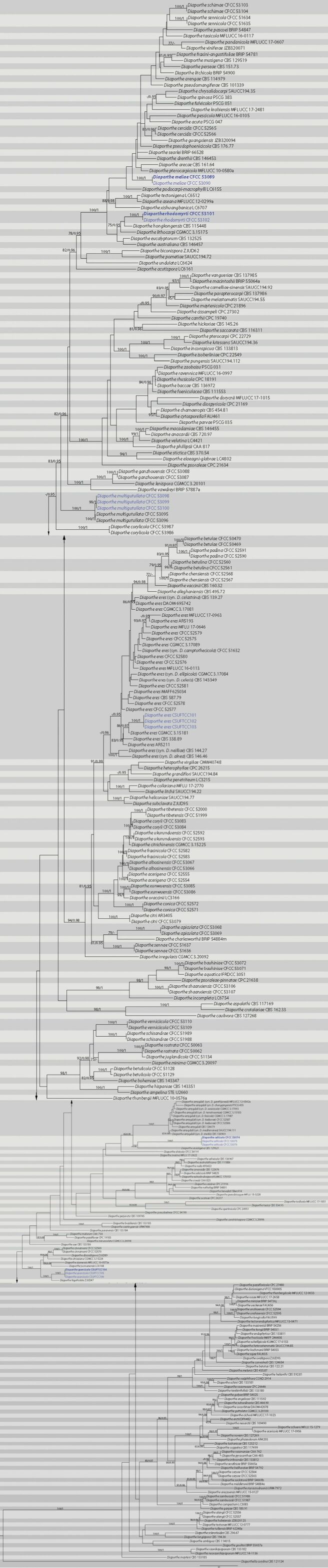
Phylogram of *Diaporthe* resulting from a maximum likelihood analysis based on combined ITS, *cal*, *his3*, *tef-1α* and *tub2*. The tree is rooted with *Diaporthellacorylina*. Values above the branches indicate Maximum Likelihood bootstrap (left, ML BP ≥ 75%) and Bayesian probabilities (right, BI PP ≥ 0.95). Strains in current study are in blue and the ex-type cultures are in bold.

The sequence datasets for the ITS, *cal*, *his3*, *tef-1α* and *tub2*, were analysed in combination to infer the interspecific relationships within *Diaporthe*. The combined species phylogeny of the *Diaporthe* isolates consisted of 303 sequences, including the outgroup *Diaporthellacorylina* (CBS 121124). A total of 2535 characters including gaps (512 for ITS, 524 for *cal*, 525 for *his3*, 463 for *tef-1α*, and 511 for *tub2*) were included in the phylogenetic analysis. Similar tree topologies were obtained by ML and BI methods, and the best scoring ML tree is shown in Fig. 1. The ML analysis yielded a tree with a likelihood value of ln: -76822.498401 and the following model parameters: alpha: 0.508079; Π(A): 0.214617, Π(C): 0.326518, Π(G): 0.235187 and Π(T): 0.223678. The phylogenetic tree inferred from the concatenated alignment resolved the sixteen *Diaporthe* isolates from branch cankers or leaf spots into six well-supported monophyletic clades that represent four novel species and two known species of *Diaporthe* (Fig. 1).

### ﻿Taxonomy

#### ﻿ Diaporthe celticola

Taxon classificationFungiDiaporthalesDiaporthaceae

C.M. Tian & Q. Yang sp. nov.

94BA6A49-9023-5F28-A5A1-EA8DAC7A2EEB

 832920

Fig. 2

##### Diagnosis.

Distinguished from the other *Diaporthe* species based on DNA sequence data and characterised by conidiomata with single necks erumpent through the host bark.

**Figure 2.**
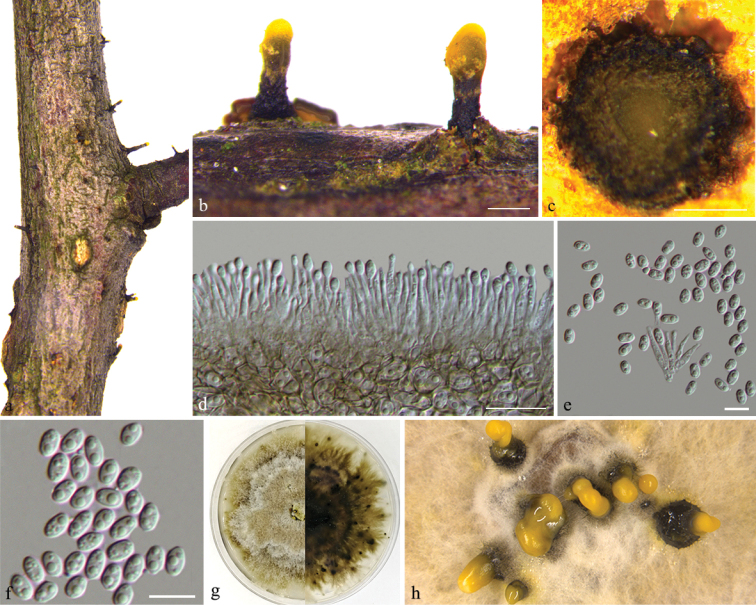
*Diaporthecelticola* (BJFC-S1616) **a, b** habit of conidiomata on twig **c** transverse section through conidiomata **d, e** conidiogenous cells with alpha conidia **f** alpha conidia **g, h** conidiomata formed on PDA. Scale bars: 200 μm (**b, c**); 10 μm (**d–f**).

##### Etymology.

Named after the host genus on which it was collected, *Celtis*.

##### Description.

***Conidiomata*** pycnidial, 535–605 × 210–225 μm diam, solitary and with single necks erumpent through host bark. ***Ectostromatic disc*** brown, one ostiole per disc, with yellowish cream conidial drops exuding from the ostioles. Tissue around the neck is cylindrical. ***Locule*** circular, undivided, 350–375 μm diam. ***Conidiophores*** reduced to conidiogenous cells. ***Conidiogenous cells*** unbranched, straight or sinuous, apical or base sometimes swelling, (8–)10.5–13(–14.5) × 1–1.5 μm (n = 30), L/W = 8.5–10.5. ***Alpha conidia*** hyaline, aseptate, ellipsoidal, biguttulate, (5–)6–7 × 3.5–4 μm (n = 30), L/W = 1.5–1.8. ***Beta conidia*** not observed.

##### Culture characters.

Colony originally flat with white fluffy aerial mycelium, becoming light brown to olive-green mycelium with age, marginal area irregularly, with yellowish cream conidial drops exuding from the ostioles.

##### Specimens examined.

China, Zhejiang Province: Hanzhou City, on branches of *Celtisvandervoetiana*, 12 May 2018, *Q. Yang* & *Y.M. Liang* (holotype BJFC-S1616; ex-type living culture: CFCC 53074; living cultures: CFCC 53075 and CFCC 53076).

##### Notes.

Three strains representing *Diaporthecelticola* cluster in a well-supported clade (ML/BI = 100/1), and appear closely related to *D.acaciigena*. *Diaporthecelticola* can be distinguished based on ITS, *cal*, *his3*, *tef-1α*, and *tub2* loci from *D.acaciigena* (29/473 in ITS, 68/442 in *cal*, 53/460 in *his3*, 79/330 in *tef-1α*, and 49/415 in *tub2*). Morphologically, *D.celticola* is characterised by conidiomata with single necks erumpent through the host bark and can be distinguished from *D.acaciigena* by smaller alpha conidia (6–7 × 3.5–4 vs. 10–11 × 6–6.5 μm) (Crous et al. 2011). This is the first occasion that *Diaporthe* species have been discovered from infected branches on *Celtisvandervoetiana* and demonstrates it to be a new species based on phylogeny and morphology.

#### ﻿ Diaporthe eres

Taxon classificationFungiDiaporthalesDiaporthaceae

Nitschke, Pyrenomyc. Germ. 2: 245, 1870.

16F58E6D-812C-51C4-A02E-44480AAF2D26

##### Description.

See Udayanga et al. (2014b).

##### Specimens examined.

China. Beijing: Pinggu District, on branches of Populus×xiaohei, 10 July 2020, *Q. Yang* (CSUFT101; living cultures: CSUFTCC101, CSUFTCC102, and CSUFTCC103).

##### Notes.

*Diaportheeres* is the type species of the genus and was originally described by Nitschke (1870), from *Ulmus* sp. in Germany, which has a widespread distribution and a broad host range as an endophyte or saprobe, or pathogen causing leaf spots, stem cankers and diseases of woody plants (Udayanga et al. 2014b). In the present study, three isolates (CSUFTCC101, CSUFTCC102, and CSUFTCC103) are embedded into the *D.eres* species based on DNA sequence data (Fig. 1). We therefore describe *D.eres* as a known species for this clade.

#### ﻿ Diaporthe meliae

Taxon classificationFungiDiaporthalesDiaporthaceae

C.M. Tian & Q. Yang sp. nov.

E03A0E1D-05B5-58C3-855F-A510661CECCE

 829523

Fig. 3

##### Diagnosis.

Distinguished from the phylogenetically closely-related species, *D.podocarpi-macrophylli*, in shorter alpha conidia.

**Figure 3.**
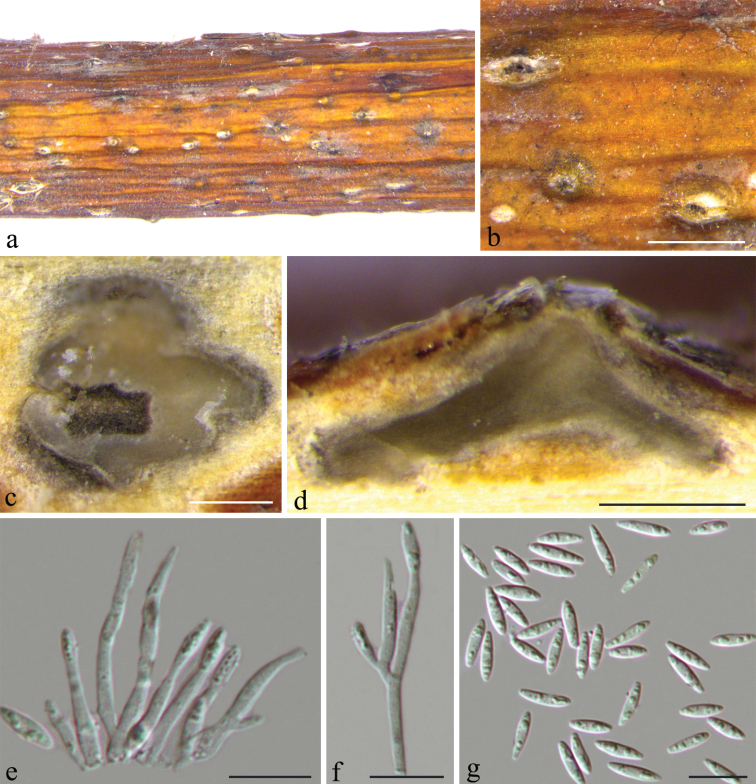
*Diaporthemeliae* (BJFC-S1668) **a, b** habit of conidiomata on twig **c** transverse section through conidiomata **d** longitudinal section through conidiomata **e, f** conidiogenous cells **g** alpha conidia. Scale bars: 1 mm(**b**); 200 μm (**c, d**); 10 μm (**e–g**).

##### Etymology.

Named after the host genus on which it was collected, *Melia*.

##### Description.

***Conidiomata*** pycnidial, immersed in the host bark, scattered, erumpent through the bark surface, discoid, with a single locule. ***Ectostromatic disc*** dark brown, one ostiole per disc, (325–)330–375(–385) μm (n = 30) diam. ***Locule*** undivided, 420–640 × 385–515 μm (n = 30). ***Conidiophores*** reduced to conidiogenous cells. ***Conidiogenous cells*** (13.5–)15–26.5(–28) × 1.3–2.1(–2.3) μm (n = 30), L/W = 8.5–15.5, cylindrical, hyaline, branched, straight or slightly curved, tapering towards the apex. ***Alpha conidia*** hyaline, aseptate, fusiform, multi-guttulate, (6.7–)8–9.5(–10) × (2–)2.1–2.3 μm (n = 30), L/W = 3.4–4.5. ***Beta conidia*** not observed.

##### Culture characters.

Colony originally flat with white felty aerial mycelium, becoming auburn furcate mycelium with age, with irregular margin, conidiomata absent.

##### Specimens examined.

China, Shandong Province: Rizhao City, on branches of *Meliaazedarach*, 20 April 2018, *N. Jiang* (holotype BJFC-S1668; ex-type living culture: CFCC 53089; living culture: CFCC 53090).

##### Notes.

Two strains representing *Diaporthemeliae* cluster in a well-supported clade (ML/BI = 100/1), and appear closely related to *D.podocarpi-macrophylli*. *Diaporthemeliae* can be distinguished based on ITS, *his3*, *tef-1α*, and *tub2* loci from *D.podocarpi-macrophylli* (4/459 in ITS, 15/455 in *his3*, 25/349 in *tef-1α*, and 14/401 in *tub2*). Morphologically, *D.meliae* can be distinguished from *D.podocarpi-macrophylli* by its longer conidiogenous cells (15–26.5 vs. 6–18 μm) and alpha conidia (8–9.5 vs. 3.5–8.5 μm) (Gao et al. 2017).

#### ﻿ Diaporthe multiguttulata

Taxon classificationFungiDiaporthalesDiaporthaceae

F. Huang, K.D. Hyde & Hong Y. Li, Fungal Biology 119(5): 343, 2015.

727D172F-F193-55A4-8849-798DC38C9D49

Fig. 4

##### Description.

See Yang et al. (2021a).

##### Specimens examined.

China, Jiangxi Province: Ganzhou City, on branches of *Citrusmaxima*, 11 May 2018, *Q. Yang*, *& Y.M. Liang* (BJFC-S1615; living cultures: CFCC 53098, CFCC 53099, and CFCC 53100).

**Figure 4.**
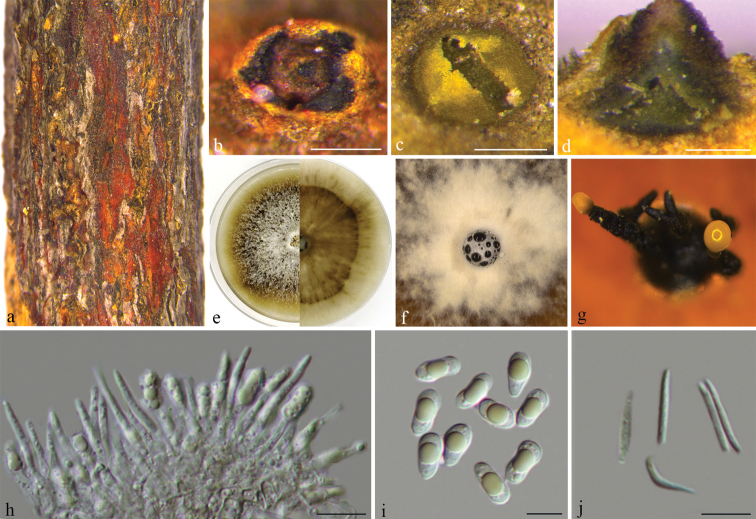
*Diaporthemultiguttulata* (BJFC-S1615) **a, b** habit of conidiomata on twig **c** transverse section through conidiomata **d** longitudinal section through conidiomata **e–g** conidiomata formed on PDA**h** conidiogenous cells **i** alpha conidia **g** beta conidia. Scale bars: 200 μm (**b–d**); 10 μm (**h–j**).

##### Notes.

*Diaporthemultiguttulata* is characterised by ellipsoidal alpha conidia with one large guttulate, and was originally described as an endophyte from healthy branch of *Citrusgrandis* in Fujian Province, China (Huang et al. 2015). Yang et al. (2021a) identified three isolates from *Citrusmaxima* as *D.multiguttulata* based on DNA sequence data and confirmed from the morphological characters. In the present study, isolates (CFCC 53098, CFCC 53099, and CFCC 53100) from an additional specimen were observed and supplemented with beta conidia (Fig. 4j).

#### ﻿ Diaporthe quercicola

Taxon classificationFungiDiaporthalesDiaporthaceae

Q. Yang sp. nov.

0BC8A009-87F3-598E-AD50-335F762CA516

 843494

Fig. 5

##### Diagnosis.

Distinguished from the phylogenetically closely-related species, *D.biguttulata*, by its filiform, eguttulate alpha conidia.

**Figure 5.**
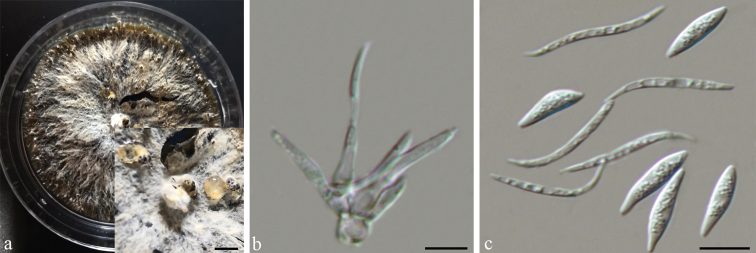
*Diaporthequercicola* (CSUFTCC104) **a** conidiomata formed on PDA**b** conidiogenous cells **c** alpha and beta conidia. Scale bars: 200 μm (**a**); 10 μm (**b, c**).

##### Etymology.

Named after the host genus on which it was collected, *Quercus*.

##### Description.

On PDA: ***Conidiomata*** pycnidial, 250–330 μm diam, globose, solitary or aggregated, deeply embedded in the medium, erumpent, single or clustered in groups of 3–5 pycnidia, coated with hyphae, cream to yellowish translucent conidial droplets exuded from the ostioles. ***Conidiophores*** reduced to conidiogenous cells. ***Conidiogenous cells*** hyaline, cylindrical, unbranched, straight, tapering towards the apex, (17–)20–26(–34.5) × 2.5–3.5 μm (n = 30), L/W = 6.5–9. ***Alpha conidia*** (6.5–)7–8.5(–9) × (1.5–)2–3 μm (n = 30), L/W = 3–4.5, aseptate, hyaline, fusiform, apex at both ends, eguttulate. ***Beta conidia*** hyaline, aseptate, filiform, straight or sinuous at one end, eguttulate, (21.5–)25.5–31(–33) × 1 µm (n = 30), L/W = 22.5–31.5.

##### Culture characters.

Colony at first white, becoming dark brown with age. Aerial mycelium white, dense, fluffy, with yellowish conidial drops exuding from the ostioles after 20 days.

##### Specimens examined.

China. Shaanxi Province: Xian City, on branches of *Quercusaliena*, 10 July 2020, *Q. Yang* (holotype CSUFTCC104; ex-type living culture: CSUFTCC104; living cultures: CSUFTCC105 and CSUFTCC106).

##### Notes.

Three strains representing *Diaporthequercicola* cluster in a well-supported clade (ML/BI = 100/1), and appear closely related to *D.biguttulata*. *Diaporthequercicola* can be distinguished based on ITS, *his3*, and *tef-1α* loci from *D.biguttulata* (8/461 in ITS, 18/448 in *his3*, and 22/325 in *tef-1α*). Morphologically, *D.quercicola* can be distinguished from *D.biguttulata* by its fusiform, eguttulate alpha conidia and narrower beta conidia (1 vs. 0.9–1.6 μm) (Huang et al. 2015).

#### ﻿ Diaporthe rhodomyrti

Taxon classificationFungiDiaporthalesDiaporthaceae

C.M. Tian & Q. Yang sp. nov.

BA16FB6D-96D7-5682-BB59-CDD569F6B6BA

 829525

Fig. 6

##### Diagnosis.

Distinguished from the phylogenetically closely-related species, *D.hongkongensis*, in narrower beta conidia.

**Figure 6.**
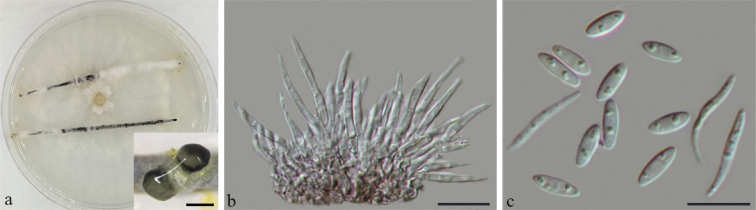
*Diaportherhodomyrti* (BJFC-S1660) **a** conidioma formed on PNA**b** conidiogenous cells **c** alpha and beta conidia. Scale bars: 500 μm (**a**); 10 μm (**b, c**).

##### Etymology.

Named after the host genus on which it was collected, *Rhodomyrtus*.

##### Description.

On PNA: ***Conidiomata*** pycnidial, 500–850 μm diam, globose or rostrate, black, erumpent in tissue, erumpent at maturity, often with translucent conidial drops exuding from ostioles. ***Conidiophores*** reduced to conidiogenous cells. ***Conidiogenous cells*** (14.5–)15.5–23(–25.5) × 1.5–2 μm (n = 30), L/W = 8.5–13, cylindrical, hyaline, unbranched, septate, straight, tapering towards the apex. ***Alpha conidia*** abundant in culture, hyaline, aseptate, ellipsoidal, biguttulate, 6–7(–8.5) × 2–2.5(–3) μm (n = 30), L/W = 2.8–3.3. ***Beta conidia*** hyaline, aseptate, filiform, straight to sinuous, eguttulate, (15–)16.5–21.5(–23) × 1–1.5 µm (n = 30), L/W = 15.5–16.5.

##### Culture characters.

Colony entirely white at surface, reverse with pale brown pigmentation, white, fluffy aerial mycelium.

##### Specimens examined.

China. Jiangxi Province: Ganzhou City, on leaves of *Rhodomyrtustomentosa*, 10 May 2018, *Q. Yang* & *Y.M. Liang* (holotype BJFC-S1660; ex-type living culture: CFCC 53101; living culture: CFCC 53102).

##### Notes.

This new species is introduced as molecular data, and shows it to be a distinct clade with high support (ML/BI = 100/1) and appears closely related to *Diaporthehongkongensis*. *Diaportherhodomyrti* can be distinguished based on ITS, *cal*, *his3*, *tef-1α*, and *tub2* loci from *D.hongkongensis* (2/463 in ITS, 26/441 in *cal*, 11/434 in *his3*, 10/327 in *tef-1α*, and 2/420 in *tub2*). Morphologically, *D.rhodomyrti* can be distinguished from *D.hongkongensis* by its longer conidiogenous cells (15.5–23 vs. 5–12 μm) and narrower beta conidia (1–1.5 vs. 1.5–2 μm) (Gomes et al. 2013). This is the first time that *Diaporthe* species has been discovered from infected leaves on *Rhodomyrtustomentosa* and demonstrate it as a new species based on phylogeny and morphology.

## ﻿Discussion

In this study, investigations of forest pathogens in Beijing, Jiangxi, Shaanxi and Zhejiang Provinces was carried out. Identification of our collections was conducted, based on isolates from fruiting bodies using five combined loci (ITS, *cal*, *his3*, *tef-1α*, and *tub2*), as well as morphological characteristics. It includes *Diaportheeres* and *D.multiguttulata*, as well as four new species named *D.celticola*, *D.meliae*, *D.quercicola*, and *D.rhodomyrti*.

*Diaporthe* (Diaporthaceae, Sordariomycetes) are species-rich asexual taxa, which are common pathogens that cause a variety of diseases, including dieback, stem cankers, leaf spots, leaf and pod blights, fruit rots and seed decay (Uecker 1988; Rehner and Uecker 1994; Mostert et al. 2001; Thompson et al. 2001; Santos et al. 2011). Because many *Diaporthe* species have overlapping morphological traits, sequence data is essential to resolve this genus and introduce new species (Udayanga et al. 2014a). Combined gene sequence of ITS, *cal*, *his3*, *tef-1α*, and *tub2* is the optimal combination for species delimitation (Santos et al. 2017). However, removing the ITS locus has little effect on reconstructed phylogenies, identifying the *cal-his3-tef-1α-tub2* four loci tree as almost equivalent to the five loci phylogenetic tree.

Many confusions occur in species separation of *Diaportheeres* complex with the lack of an ex-type culture or ex-epitype culture, although a broad species concept has historically been associated with *D.eres* (Udayanga et al. 2014b). Fan et al. (2018) demonstrated the effectiveness of three loci, including *cal*, *tef-1α* and *tub2*, for the identification of the *D.eres* complex in walnut trees. Similarly, Yang et al. (2018) also used three-locus sequences to identify *D.eres* species associated with different hosts in China, and Chaisiri et al. (2021) revealed the phylogenetic analysis from the combined dataset of *cal*, *his3*, *tef-1α* and *tub2* was highly effective, but the ITS region impeded species delimitation, which conforms with Yang et al. (2018).

Recently, several studies have been conducted associated with various hosts in China. For instance, the research conducted by Guo et al. (2020) revealed six novel *Diaporthe* species that infect pears and are responsible for pear shoot canker. Sun et al. (2021) showed high species diversity of *Diaporthe* in tropical rain forests, with description of eight new species. Wang et al. (2021) represented the first characterization of *Diaporthe* species associated with peach constriction canker in China, and contributed useful data for practicable disease management. Yang et al. (2021b) identified two new species from *Camelliaoleifera*, which is an important edible oil woody plant in southern China. This study also characterises the taxonomic and morphological diversity of *Diaporthe* spp. associated with different hosts, which indicated there is a potential of *Diaporthe* species remains to be discovered in China.

## Supplementary Material

XML Treatment for Diaporthe celticola

XML Treatment for Diaporthe eres

XML Treatment for Diaporthe meliae

XML Treatment for Diaporthe multiguttulata

XML Treatment for Diaporthe quercicola

XML Treatment for Diaporthe rhodomyrti
